# The Poor, Our Voluntary Hospitals and Nurses

**Published:** 1912-12-21

**Authors:** 


### THE POOR, OUR VOLUNTARY HOSPITALS AND NURSES.

### By the Right Rev. the Lord Bishop of London.

I look upon it as a real privilege to be allowed to stand here* for a few moments and speak a word of encouragement, or what my friend calls the blessing of the Church, to those who have toiled so long and given so much in the cause of suffering humanity. And I want to direct my remarks to what I know better than many of you, the contrast between the places where we find the patients, and the position they are in when they carry them into the hospitals. Therefore I shall not speak to-day so much as Bishop of London as I shall as an old worker for nine years, who has been moving up and down in the slums of East London, and who saw by personal experience what the hospitals meant in the lives of the suffering poor. On your afternoon visit you see some poor fellow lying without a bed, on a heap of rags. Look at the difference between that man's surroundings and, if you can persuade him to go, what he gets in the hospital. Look at him two or three hours afterwards, when he is comfortably lying on a good bed in one of the London hospitals. It is almost the difference between Hell and Heaven. And what a difference to the chance of life that man has compared with the condition in which you found him. Perhaps, as I have found often, there is a family, consisting of father and mother and six children, in one room—eating, sleeping, and living. Imagine that person transplanted from the awful atmosphere of that room into the clean ward of a London hospital, with nurses and doctors attending him close at hand. Just picture the difference between the man's chance of life which he had in one place, and compare it with that in the other. It is true that twenty-four years ago, when I started work of this kind, we had to persuade them that they were not going to be experimented on in the interests of science. That took some little time. But these people had so many uncles and aunts, and fathers and mothers, and brothers and sisters who came back to give a good account of what they had in the hospital that they now look upon it as, to use one common expression in Bethnal Green, "a bit of all right." Not only is the contrast so great, but when you visit these hospitals, what a scene they are of happy, useful activity! You who do not know them may think of them as miserable places. I say there never could be a brighter place than a ward in a London hospital. It is true there is that little shrouded bed which you see in the corner with the curtains drawn round, indicating that some soul is about to pass soon, in all probability, from this world to the other, and you notice the voluntary silence and quietude of that ward, and it shows what good-hearted people our people are after all, and how they enter into the sorrows of one another. Take the accident ward in a London hospital. It is one of the brightest scenes you can go into. For nine years, every Monday, I took about twenty young officers and men who were working with me into the London Hospital. I picture just now the old man in the corner, who is the subject of mild chaff among the others, which he greatly enjoys; and Tommy, who is recovering from his accident, taking round the tea; and the broken-legged man in the corner having the time of his life, feeding like a fighting-cock, and having a six weeks' rest in his bed, which he has never had in his life before and probably will never have again. Therefore, if you want a picture of bright activity and helpfulness, go to the ward of a London hospital. And when Christmas comes, what a time it is then! It is certainly worth while breaking your leg, or your collar-bone, or or even an arm, to have all the joys of Christmas in a voluntary hospital. There are the toys and the Christmas trees, and the concerts, nurses and doctors doing all they can to make Christmas bright and happy. You may say that is all on the surface. I admit that what we do see is on the surface, as far as that goes. But let us look below the surface. See what it means to keep these great lifeboats on the sea of suffering really at work day and night, as they are kept. The first thing it means is really beautiful unpaid work on the part of the great surgeons and physicians who are what we call "on the staff." There are noble traditions about this work—men proud of their country, men sought after, who are occupying these honorary posts in the great hospitals, and absolutely unpaid. These men are giving their work, and what the rich man has to pay a fee of £100 for, for his operation, is given to these hospital patients with as much skill and care, and for nothing. That means great vigilance and great responsibility on the part of those house surgeons and physicians, who are chosen, as you know, from the most promising of those who become qualified from among the students. In my first curacy I was honorary chaplain to a great county infirmary, and I made friends with the young house surgeon. And I was greatly interested in his first operation, tracheotomy, and—this will show you the kind of spirit these men work in—I know he walked fourteen miles out into the country to see how his little patient was going on whom he had operated on in the hospital. And then there is the wonderful devotion and skill and hard work of the sisters and nurses. It is a hard life, let us admit that, and I think we should encourage no young woman whom we know, who is not fairly strong, to attempt the life. But what a reward they have in the gratitude of their patients on account of the amount of suffering and misery which they relieve, and the happiness they bring. It is not only a hard life, but there are those who say it makes them hard. But after twenty-four years' knowledge of hospitals, I have not seen that. But I can bring back a scene to my memory, one especially, of a lad of twenty-two who

* At St. James's Palace, where this speech was delivered to the League of Mercy on Dec. 16.

was fading away from this world in the arms of his mother, and I glanced round, and while every nurse was at her post, and not a hand quivered, there was not a dry eye in the ward as that man died. These nurses have to be absolutely self-controlled, and they have to be, as we should expect them to be, perfectly trained, so as not to allow their feelings to get the better of them. But I do not believe that there are any softer or more womanly hearts in the world than those of these nurses. Without these doctors and nurses, of course, this great work in the hospitals could not be carried on, and towards this ceaseless activity the League of Mercy has been a faithful and great helper. I wish all these hospitals of ours to be always carried on by voluntary contributions, and for three reasons. The first is because it is so good for us, so good to exercise the true doctrine of stewardship, to use in such a good way the things which we possess. I have had to do with many things besides hospitals, and one of the things I did was to argue, Sunday after Sunday, against those who did not believe in God, those who opposed my beliefs in public and private, in parks, in halls, and at street corners. The one argument they used which I found it difficult to meet was the argument based on the suffering of the world. And you will be interested to know how keenly these subjects are debated. One man would say there was no God, and he would show no signs of caring. Another would be a Theosophist, and say these people have suffered in a previous state of existence, and so on, though what comfort there was in that I do not know. Then there was the keen, eager Socialist, under the red flag, with his belief that Society should be reconstructed, and should be started all over again. As a Christian I never agreed with these three lines of argument, but I was driven to this, and I think anyone would be driven to this, whether Christian or Jew—and we owe a tremendous amount to the Jews for the way they support the Fund, and I am now speaking as one holding common ground between those two religions—how can one possibly justify a belief in a loving God except by believing in the doctrine of stewardship, that we, the minority, have these things in trust, on the understanding that we pass them on to others? I like to take as an illustration two boys being sent by their father to school. The father sends them both together, and gives the elder boy the money and food for the journey. That elder boy is not doing his duty if he leaves his younger brother when he gets to Willesden Junction. This simile will show you what I consider is the function of the League of Mercy. It shows that we should be passing on what we have received to others, it is a great means of voluntary help and voluntary service. It enlarges the heart, and expands the will and the whole being. What expands the life more than living it out in personal service and discharging the stewardship in regard to what has been given to us? And what a bond of union it is between us. During the old Chief Rabbi's life he was a personal friend of mine, and we always sat side by side at the meetings of the King's Hospital Fund, and it is such a bond of union between Jews and Christians, and people of all denominations of Christians in this beautiful link of common service which is so admirable. And then let me say—and it shall be my last word—it stirs the sympathy and help of the working man. Every Hospital Sunday, when I was in East London, I used to stand in my stand in the Park and collect for the great hospitals of London. We had various little schemes. Every wearer of a top-hat had to put in gold. There were not many top-hats, as you can imagine. Then I would put in a half-crown and ask for a silver collection. But it was the copper collection which I should have liked you to see. It came like a brown rain on to the sheet, and we never collected less than £50 before tea. The voluntary hospital appeals to the best nature, to the sympathies and the co-operation of the working men of this country, and it does real good to them to feel that they can contribute to the hospitals which do so much for their kith and kin. It is therefore with a full heart, and wishing you God-speed in your excellent work, that I say:

> The quality of mercy is not strain'd;
> it is twice blest;
> It blesseth him that gives and him that takes; and I do believe that if in the next year you preach this gospel to others, and pass on the doctrine of the steward, and get others to give with something like the generosity of the Eternal Giver, then with a good conscience, with expanded sympathy and enriched life, you will have "good measure, pressed down, shaken together and running over," you will receive good back unto yourselves.

